# Benzoic Acid Derivatives with Trypanocidal Activity: Enzymatic Analysis and Molecular Docking Studies toward *Trans*-Sialidase

**DOI:** 10.3390/molecules22111863

**Published:** 2017-10-30

**Authors:** Muhammad Kashif, Antonio Moreno-Herrera, Juan Carlos Villalobos-Rocha, Benjamín Nogueda-Torres, Jaime Pérez-Villanueva, Karen Rodríguez-Villar, José Luis Medina-Franco, Peterson de Andrade, Ivone Carvalho, Gildardo Rivera

**Affiliations:** 1 Laboratorio de Biotecnología Farmacéutica, Centro de Biotecnología Genómica, Instituto Politécnico Nacional, Boulevard del Maestro, s/n, Esq. Elías Piña, Reynosa 88710, Mexico; chkashif987@gmail.com (M.K.); amorher9@hotmail.com (A.M.-H.); 2 Laboratorio de Bioquímica Microbiana, Departamento de Microbiología, Escuela Nacional de Ciencias Biológicas, Instituto Politécnico Nacional, Ciudad de México 11340, Mexico; jvillalobosr@ipn.mx; 3 Departamento de Parasitología, Escuela Nacional de Ciencias Biológicas, Instituto Politécnico Nacional, Ciudad de México 11340, Mexico; bnogueda@yahoo.com; 4 Departamento de Sistemas Biológicos, División de Ciencias Biológicas y de la Salud, UAM-X, Ciudad de México 04960, Mexico; jpvillanueva@correo.xoc.uam.mx (J.P.-V.); qkarenrodv@hotmail.com (K.R.-V.); 5 Departamento de Farmacia, Facultad de Química, Universidad Nacional Autónoma de México, México City 04510, Mexico; medinajl@unam.mx; 6 School of Pharmaceutical Sciences of Ribeirão Preto, University of São Paulo, Av. Café s/n, Ribeirão Preto SP 14040-930, Brazil; petersondeandrade32@gmail.com (P.d.A.); carronal@usp.br (I.C.)

**Keywords:** benzoic acid, Chagas disease, docking, inhibitors, *trans*-sialidase

## Abstract

Chagas, or American trypanosomiasis, remains an important public health problem in developing countries. In the last decade, *trans*-sialidase has become a pharmacological target for new anti-Chagas drugs. In this work, the aims were to design and find a new series of benzoic acid derivatives as *trans*-sialidase (TS) inhibitors and anti-trypanosomal agents. Three compounds (**14**, **18**, and **19**) sharing a *para*-aminobenzoic acid moiety showed more potent trypanocidal activity than the commercially available drugs nifurtimox and benznidazole in both strains: the lysis concentration of 50% of the population (LC_50_) was <0.15 µM on the NINOA strain, and LC_50_ < 0.22 µM on the INC-5 strain. Additionally, compound **18** showed a moderate inhibition (47%) on the *trans*-sialidase enzyme and a binding model similar to DANA (pattern A).

## 1. Introduction

Chagas disease, or American trypanosomiasis, is a chronic disease caused by the kinetoplastid protozoan parasite *Trypanosoma cruzi*. Although this disease is present mostly in the endemic poor rural areas of America (from Southern California to Argentina), it is also becoming an important health issue in metropolitan areas [1] and other non-endemic areas, such as North America [2] and Europe [3,4], due to population migrations [5,6,7,8,9,10,11]. In fact, the World Health Organization (WHO) has estimated that Chagas disease is nowadays affecting about 7 to 8 million people worldwide [12] and accounting for the death of over 12,500 patients per year [13,14].

Strategies to manage Chagas disease are based fundamentally on the regional elimination of domestic vectors [15] and on the pharmacological treatment of patients. According to chemotherapy treatment, the nitroheterocyclic compounds benznidazole (*N*-benzyl-2-(2-nitro-1*H*-imidazole-1-yl)acetamide, Bnz) and nifurtimox (3-methyl-*N*-[(5-nitrofuran-2-yl)methylidene]thiomorpholin-4-amine-1,1-dioxide, Nfx), launched by Bayer in 1967 and Roche in 1972, respectively, are the only two drugs currently used for the treatment of Chagas disease, although they are not effective against its chronic form [16], diminish in efficacy the longer a person has been infected [12], and the treatment is associated with severe toxic side-effects [17,18,19,20,21,22]. In order to address this major health issue, new anti-Chagas drugs directed to different targets are being designed in order to modulate relevant enzyme activities in biochemical pathways, such as cysteine proteases, *trans*-sialidase, and trypanothione reductase [23].

*Trypanosoma cruzi trans*-sialidase (TcTS) is a potential target for Chagas disease chemotherapy. The surface of invasive *T. cruzi* trypomastigotes is covered by numerous *trans*-sialidases, whose main role is to acquire sialic acid units from mammalian host glycoconjugates and transfer them to the parasite membrane generating α-2,3-sialylated-β-galactopyranose units and providing (i) direct protection from recognition by the host’s immune system [24], and (ii) resistance to the complement and immediate survival of trypomastigotes released to the bloodstream [25]. TcTS also aids in the recognition of and attachment to host cells through active site-mediated binding to sialic acids and/or β-galactosyl residues on the surface of host cells [26,27,28,29,30] or through domains distinct from the active site [31,32].

**Figure 1:**
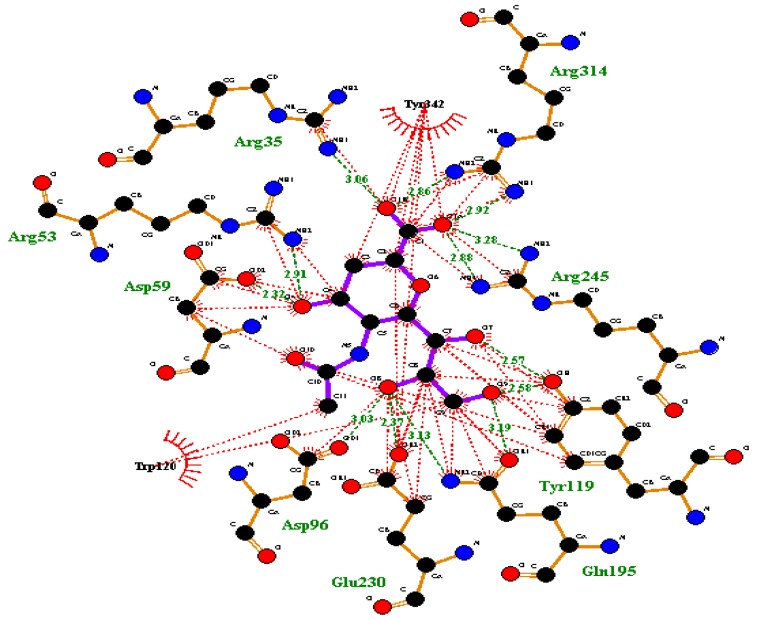
Sialic acid analogous 2,3-dehydro-3-deoxy-*N*-acetylneuraminic acid (DANA) interaction on active sites of *Trypanosoma cruzi trans*-sialidase (TcTS) enzyme (green line showing a conventional hydrogen bonding interaction and red lines showing a hydrophobic interaction). The image was produced with LigPlot+ software (v.1.4, European Bioinformatics Institute (EMBL-EBI), Hinxton, Cambridge, UK).

The active site of TcTS contains several conserved microbial sialidase features showing a donor site related to sialic acid interaction and an acceptor site associated with the β-galactose molecule. The donor site contains an arginine triad (Arg35, Arg245, and Arg314), which interacts with the carboxylate group (Figure 1) of sialic acid, two residues for the stabilization of the transition state (Tyr342 and Glu230), an aspartate (Asp59) essential for catalysis, and a hydrophobic pocket for accommodating sialic acid’s *N*-acetyl group. The acceptor site contains the amino acids Tyr119 and Trp312, which is crucial for the *trans*-glycosylation process, Asp59 (common to both sites), and Glu362, which directly interacts with the β-galactose acceptor [14,33].

**Figure 2:**
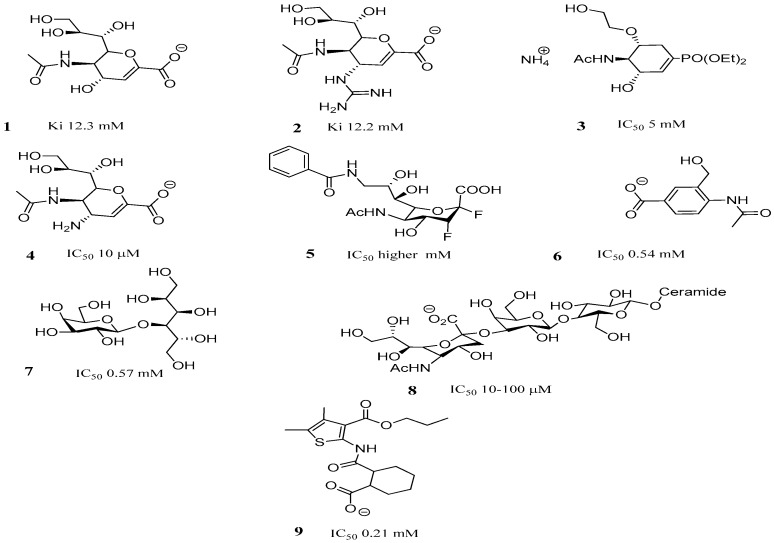
Structure of representative *trans*-sialidase inhibitors.

The inhibitors reported for TcTS have complex chemical structures. DANA, (**1**, Figure 2), a potent inhibitor of influenza neuraminidase, poorly inhibits TcTS with a reported Ki of 12.3 mM [34]. The DANA analogs **2**–**4** have exhibited improved (Ki of 12.2 mM, IC_50_ of 5 mM and 10 μM, respectively) but still weak TcTS inhibition [35,36]. The 2,3-difluorosialic acid analogs, such as **5** (Figure 2), are mechanism-based TcTS inhibitors, which form a covalent bond with the hydroxyl group of Tyr342, but only at high concentrations (mM range) [37]. Mimicking a sialic acid structure has been explored in the search for potential TcTS inhibitors. The benzoic acid derivative compound **6** (Figure 2) [35] can be highlighted, showing an IC_50_ of 0.54 mM. In the predicted docked conformation of **6** (Figure 2), its carboxylate moiety provides the expected strong interaction with the arginine triad (residues 35, 245 and 314). The hydroxyl group can form hydrogen bonds with both Glu230 and Gln195, and the amide NH group is hydrogen-bonded to Asp96, with the acetyl group located in the hydrophobic pocket. However, the apparently good fit predicted for **6** did not translate into strong TcTS inhibition [35].

Compounds such as lactitol **7** (IC_50_ = 0.57 mM) (Figure 2) and other lactose (acceptor substrate) analogues, which target the sialic acid acceptor site, were also weak inhibitors [38,39]. The most potent TcTS inhibitor known, resulting from modifications in the GM3 ganglioside (**8**, Figure 2) and with IC_50_ values in the 10–100 µM range [40], fills in both sialic acid acceptor and donor sites. This result shows the importance of designing compounds occupying both binding sites. This can clearly be observed in the docked conformation of compound **9** (MuNANA assay screening at 1 mM: 87% inhibition and IC_50_ = 0.21 mM) (Figure 2), where the respective carboxylate group interacts well with the arginine triad, and the amide function hydrogen bonds with Arg245 and Asp59, thus providing additional stabilization of the triad. The thiophene ring is seen in the sialic acid acceptor binding site and the benzene ring is positioned in the sialic acid site. The latter does not fill this site, thus the introduction of functional groups could provide further interactions with key amino acid residues (e.g., Asp96, and Gln195) [41].

**Figure 3:**
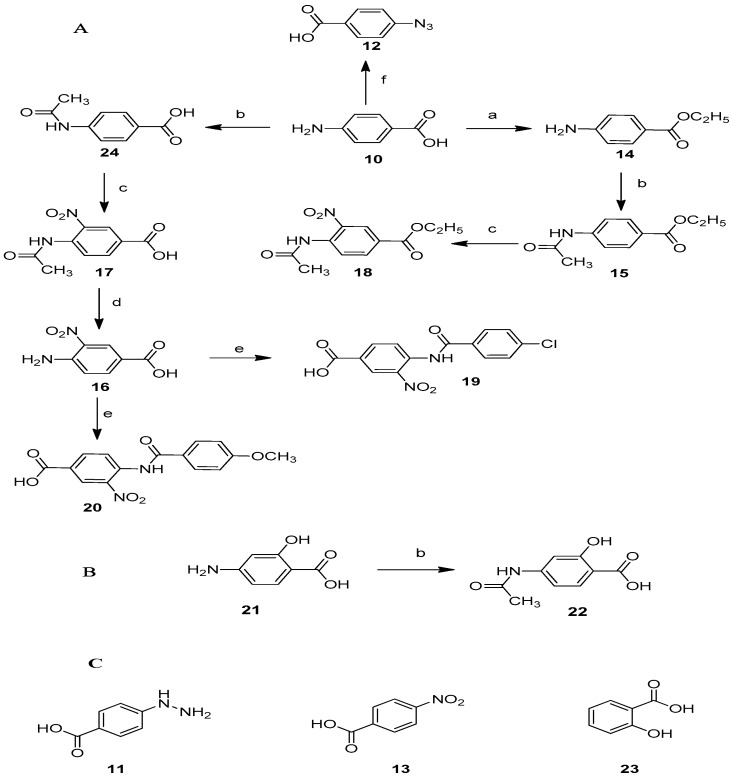
(**A**,**B**) Compounds designed and synthesized in this work. *Reagents and condition*: (a) dry CH_3_CH_2_OH, conc H_2_SO_4_, Δ; (b) CH_3_CO_2_H/(CH_3_CO)_2_O, Δ; (c) HNO_3_ and conc H_2_SO_4_, Δ; (d) H_2_SO_4_, Δ; (e) *p*-ClC_6_H_4_COCl, Et_3_N (**19**), *p*-CH_3_OC_6_H_4_COCl, Et_3_N (**20**); (f) HCl, NaNO_2_, and CH_3_COONa/NaN_3_ in H_2_O; (**C**) Compounds acquired from Sigma-Aldrich, Mexico.

In view of these previous works, and taking into account that inhibitor design against the sialidases or *trans*-sialidases of trypanosomes is reduced, our approach involved the synthesis of new non-sugar-based benzoic acid derivative inhibitors of TcTS that may lead to biological tools for or potential leads in drug design. The fact that the negatively charged carboxylate group of sialic acid represents the most important group able to interact with the sialic acid donor site through strong interactions with the arginine triad led us to design simple structures containing this group. Neres et al., 2007 synthesized *meta*- and *para*-substitutions of benzoic acid derivatives providing 4-acetylamino-3-hydroxymethylbenzoic acid (**6**, Figure 2) as the lead inhibitor, not containing the synthetically complex sialic acid structure (IC_50_ = 0.54 mM). However, the weak inhibition prompted us to synthesize new non-sugar-based benzoic acid derivative inhibitors of TcTS with better interaction with both the sialic acid donor and acceptor sites of the TcTS enzyme. Our strategy was based on a molecular simplification of these previous compounds, performing slight, but chemically sensitive changes in the *meta* and *para* positions to the carboxylate group, and at the same time, we wondered whether or not an *ortho* substituent to the carboxylate group in the aromatic ring could establish better interaction with both the sialic acid donor and acceptor sites of the TcTS enzyme. In fact, docking studies were performed to support the design of better TcTS inhibitors and showed that there is a possibility of including an *ortho* substituent to the carboxylate group in the aromatic ring, which could bind the acceptor substrate binding site, thus offering room for improvement [35]. In this context, and following the interest in our research group to develop new therapeutic options for Chagas disease, we designed new derivatives of benzoic acid with *ortho*-, *meta*-, and *para*-substitutions according to preliminary docking studies and calculated physicochemical properties (Figure 3).

## 2. Results and Discussion

### 2.1. Synthesis

Benzoic acid derivatives (**10**, **11**, **13**, **21** and **23**) [42,43] were purchased from Sigma-Aldrich. The other compounds were synthesized through the reported procedure for esterification (**14**), nitration (**17** and **18**), hydrolysis (**16**), and condensation (**15**, **19**, **20**, **22** and **24**) [44,45,46] as detailed in the Experimental Section. All compounds were obtained easily with good yields (60–90%), except compounds **19** and **20** (20–40%).

### 2.2. Trypanocidal Activity

Initially, the lytic effect of the benzoic acid derivatives and reference drugs (Nfx and Bnz) on mouse blood trypomastigotes was evaluated in vitro, using INC-5 and NINOA *T. cruzi* strains (Table 1) [47]. Furthermore, 4-acetamidobenzoic acid (**24**) previously assayed by Neres et al. [35] for TcTS inhibition has been studied.

Biological activity showed that both reference compounds Nfx and Bnz have a similar lysis concentration of 50% of the population (LC_50_) in each strain: 0.21–0.29 µM against the NINOA strain and 0.62–0.68 µM against the INC-5 strain. As shown in Table 1, amongst the tested compounds, the ethyl benzoate derivatives **14** (benzocaine, a known anesthetic agent), **15**, and **18** displayed significant trypanocidal activities in the range of 0.02–0.34 µM against the NINOA strain and 0.10–0.22 µM against the INC-5 strain, comparing to those of the corresponding carboxylic acid precursors **10**, **24**, and **17**, respectively: 0.52–1.39 µM and 0.21–1.24 µM in the NINOA and INC-5 strains, respectively. Thus, the introduction of a hydrophobic group such as ethyl showed an increase of activity. In fact, these compounds presented trypanocidal activity comparable to the reference Bzn and Nfx against the NINOA strain and significantly higher against the INC-5 strain.

Compound **19**, showing significant trypanocidal activity against the NINOA strain (0.14 µM) and the highest activity against the INC-5 strain (0.0008 µM), became a lead structure of the benzoic derivative series. The subsequent modification of this structure by replacing the chloride substituent by the acceptor methoxy group **20** did not improve the activity in both the NINOA (0.61 µM) and INC-5 (0.43 µM) strains.

Compound **10** is a simplification of the structures synthesized by Neres et al. [35] displaying moderate trypanocidal activity against the NINOA (0.52 µM) and INC-5 (1.24 µM) strains. The structure-activity relationship (SAR) of these compounds showed that the replacement of the *para*-amino group by nitro (**13**), hydrazine (**11**), or azide (**12**) groups showed comparable trypanocidal activities against the NINOA strain (0.47–0.66 µM) and an improvement against the INC-5 strain (0.46–0.58 µM). On the other hand, the *meta*-nitration of compound **10** afforded compound **16** to display a notably reduced activity against the NINOA strain (1.37 µM), whereas it increased the activity against the INC-5 strain (0.63 µM). Alternatively, *ortho*-hydroxylation afforded compound **21** to display significant trypanocidal activity against the NINOA (0.27 µM) and INC-5 (0.26 µM) strains. Compound **21** presented a higher trypanocidal activity than references Bzn and Nfx. Thus, the incorporation of a hydrophilic group as a hydroxyl in the *ortho* position of the *para*-amino benzoic acid moiety may provide a center for H-bond interactions and it might be positively affecting the biological activity. In fact, the elimination of the *para*-amino group generated compound **23** with lower trypanocidal activities (0.57 µM and 0.72 µM against the NINOA and INC-5 strains, respectively) and in addition, the *N*-acetylation of this group (compound **22**) significantly reduced the biological activity (1.28 µM in both strains). This was partially observed in compounds **24** and **17**, which did not reflect an improvement of the activity in relation to the *para*-aminobenzoic acid precursors **10** and **16** against the NINOA strain (>1.0 µM) but showed better trypanocidal activities against the INC-5 strain (0.87 and 0.21 µM, respectively). It is remarkable that a different trypanocidal activities tendency was observed in both strains. Finally, three compounds (**14**, **18**, and **19**) showed more potent trypanocidal activity than the commercially available drugs Nfx and Bnz in both strains: LC_50_ < 0.15 µM in the NINOA strain and LC_50_ < 0.22 µM in the INC-5 strain, all of them sharing the modified *para*-aminobenzoic ester moiety except **19**. These structures can be used as references for current and future studies for the synthesis of new anti-Chagas compounds. We have demonstrated that the incorporation of *ortho*-hydroxyl groups in *para*-aminobenzoic acid derivatives successfully provided a more potent compound (**21**). The esterification of the carboxylic acid (**14**, **15**, and **18**) generating a hydrophobic moiety to increase the activity could be a good strategy in these compounds. However, the introduction of a *meta*-nitro group did not initially generate a more active compound (**16**), although subsequent structural modifications finally modulated the activity and improved it. Further assays are required to understand the electronic and steric properties of *meta* and *ortho* substituents in the *para*-aminobenzoic derivative structures.

### 2.3. TcTS Inhibition

TcTS inhibition screening results, including percentage inhibition values for a series of substituted benzoic acid derivatives, are given in Table 2. The percentage inhibition at 1 mM concentration is the average of at least three independent experiments. The enzymatic inhibition assay was performed using a continuous fluorimetric method based on the TcTS-catalyzed hydrolysis of 2-(4-methylumbelliferyl)-a-d-*N*-acetylneuraminic acid (MuNANA). As a control, the activities of pyridoxal phosphate (Pyr) and compound **24** [35] were measured in the same concentrations of the target compounds due to their respective moderate [48] and weak activities on TcTS.

Pyridoxal has been reported as a non-competitive TcTS inhibitor with a Ki = 7.3 mM [49] showing a moderate inhibition (73% at 1.0 mM in the presence of MuNANA 0.1 mM). In this work, the inhibition percentage of pyridoxal phosphate was 64%, similar to previous studies [50]. On the other hand, contrary to results described by Neres et al. [35], the inhibition of compound **24** was 30% instead of 0%. These differences could be mainly associated with the enzymatic assays’ particularities. Nonetheless, this compound shows a weak inhibition, lower than pyridoxal phosphate.

According to Table 2, compounds **10**–**24** showed a variable influence on TcTS enzyme inhibition. All of the substances displayed a lower inhibitory activity against TcTS than pyridoxal, excepting compounds **11** and **17** which blocked 61 and 66% of the enzyme activity, respectively, with the same inhibitory effect as that of pyridoxal, and compound **16** showed the best inhibition activity (77%). Based on the inhibitory activity of these compounds, the *para*-amino-*meta*-nitrobenzoic acid core may have a relevant role in TcTS inhibition. Accordingly, compound **18**, obtained from the ethyl esterification of compound **17**, also showed moderate results (47%); however, esterification decreases the activity. This behavior also can be observed in compounds **10** and **24**, where the activity decreases as compared with their ethyl ester analogs **14** and **15**, respectively. The lack of the nitro substituent reduced notably the inhibition activity of these compounds. Consequently, the *ortho*-hydroxyl benzoic acid derivative compounds **21**–**23** and the *para*-amino benzoic acid/benzoate derivative compounds **10**, **14** and **15** showed weak inhibition results in the range of 1–34%. However, the replacement of the *para*-amine group of the benzoic acid by hydrazine (**11**), azide (**12**), or nitro (**13**) groups lead to moderate results, with 61, 40, and 43% of inhibition, respectively. Again, the nitro group observed in compound **13** plays an important role in the interaction with the TcTS active site. Compounds **19**–**20** were not tested due to a low solubility in DMSO 1.0% in the well.

The significant TcTS inhibition shown by compounds **11**, **16** and **17** (>60% inhibition) may direct the development of new derivatives as TcTS inhibitors, pointing out the necessity to maintain benzoic acid/benzoate core and nitrogenous moieties (amine, *N*-acetyl, nitro, azide, or hydrazine) for an efficient inhibition of this enzyme.

In summary, we can observe that there is no correlation between trypanocidal activity and TcTS inhibition. For example, of the benzoate derivatives **14**–**15** and **17**–**19** showing moderate trypanocidal activities (0.10–0.028 µM in the NINOA strain and 0.0008–0.22 µM in the INC-5 strain), only compound **15** moderately blocked the TcTS enzyme as expected with 47%, whereas the other compounds inhibited less than 7%. Nevertheless, the importance of *para*-amino carboxylic derivatives acting as TcTS inhibitors or lysing trypomastigotes via other biological mechanisms against *T. cruzi* has been demonstrated.

### 2.4. Molecular Docking

**Figure 4:**
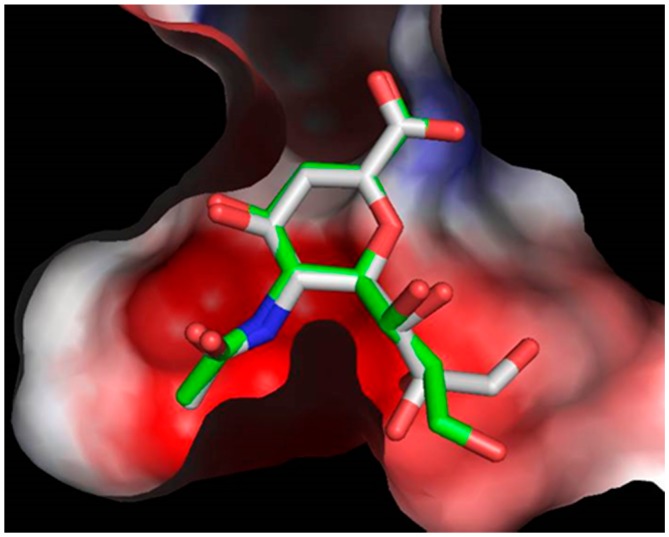
Best scored binding mode for DANA obtained with AutoDock Vina (gray) and binding mode for DANA in the original crystal structure (green). The TcTS surface is color-coded by the electrostatic potential (blue shift showing positive electrostatic potential & red shift showing negative electrostatic potential).

Docking studies were conducted for compounds **10**–**24** in order to obtain potential putative interaction of these compounds on the active site of TcTS. The docking analysis was validated using the crystal structure of TcTS against the reference inhibitor DANA [34]. The good superposition between the DANA structures oriented with AutoDock Vina 5.6 as compared with the orientation in the crystal structure (Protein Data Bank (PDB) accession number 1MS8) suggested that the chosen method is appropriate (Figure 4). The potential DANA binding site was also predicted as a deep cavity including a restricted space with two delimited regions, where the blue shift indicates a positive electrostatic potential (carboxylate interaction) and the red shift a negative electrostatic potential (amide interaction) (Figure 4).

In this study, the proposed interaction modes of the benzoic and benzoate derivatives into the active site of TcTS were determined as the highest-scored conformations (best-fit ligands), which correspond to the structure with the most favorable free energy for binding in TcTS.

**Figure 5:**
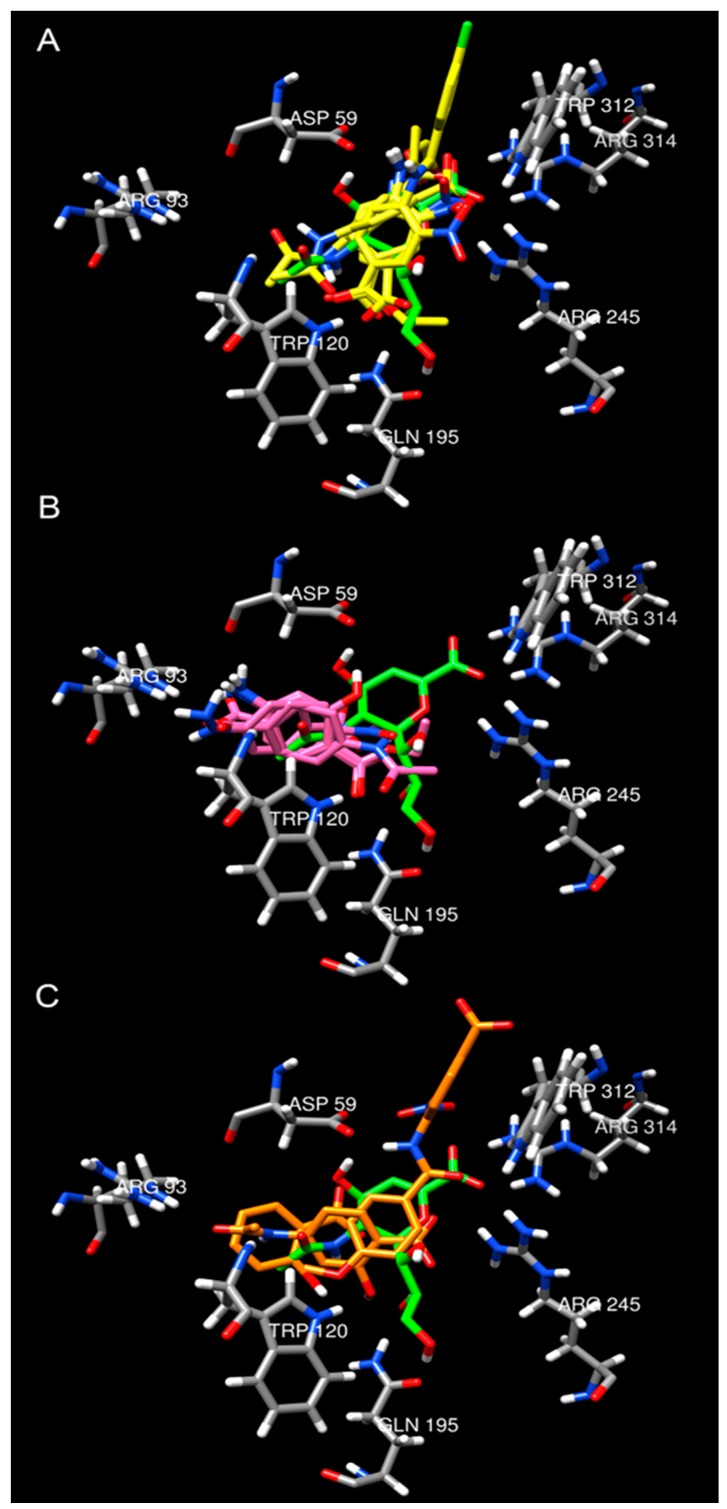
Best-scored conformations obtained for compounds **10**–**24** and their comparison with the ligand DANA in the crystal structure of TcTS (green). Compounds with binding modes classified as A are shown in pannel (**A**) as yellow structures; those classified as B are shown in pannel (**B**) as pink structures; and those classified as C are shown in pannel (**C**) as orange structures.

According to the results (Table S1, Supplementary Materials), two major binding patterns were found, A and B (Figure 5A,B). Moreover, some conformations that did not have a particular binding mode were grouped as C (Figure 5C). The first mode (A) is similar to that reported for DANA in the crystal structure (Figure 5A). Although this binding mode was found to be the best result for compounds **15**, and **24**, it does not correspond to the lowest energy conformation in most cases. It is noteworthy that, binding mode A was found to be among the best nine conformations for most of the ligands, having a good calculated affinity (Table S1). Similarly, molecules **12** and **16**–**19** showed their best-scored binding in an alternative way, designated A2 (Table S1), which is a slight variation of the binding mode A.

**Figure 6:**
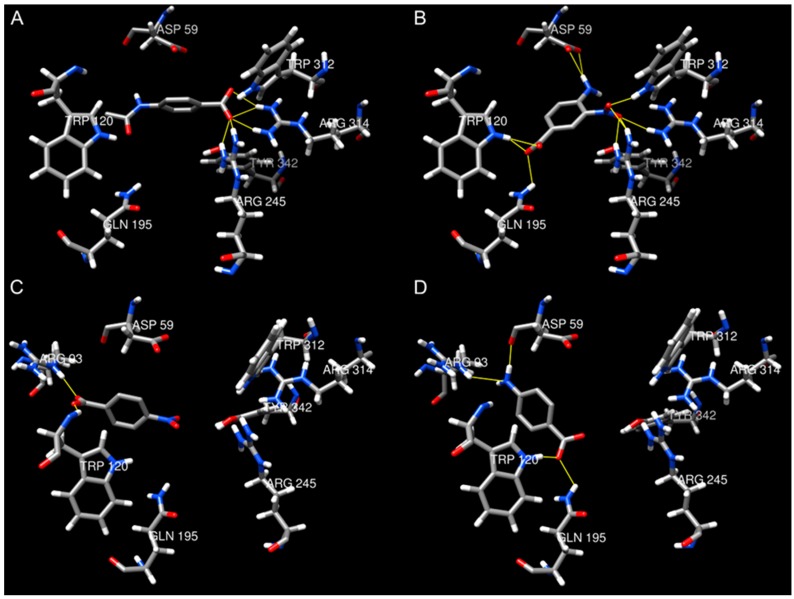
Best-scored conformations and interactions of selected compounds with TcTS. (**A**) compound **24** in binding mode A; (**B**) compound **16** in binding mode A2; (**C**,**D**) compounds **13** and **10** in binding mode B, respectively.

Figure 6A shows the best conformation for compound **24**, which corresponds to binding mode A, where the carboxylate group forms hydrogen bound interactions with Arg314 and Arg245 (donor site); these interactions can be slightly different for each ligand in binding mode A. For the molecules in binding mode A2, the nitro group adopts a similar orientation to the carboxylate group in DANA.

Figure 6B shows compound **16** in binding mode A2. It is noteworthy that, whereas the nitro group shows interactions with Arg314, Arg245, and Trp312, the carboxylate and amino groups have additional interactions with Gln195, Trp120, and Asp59, respectively. It is worth noting that all nitro benzoic acids and nitro benzoates are classified among the most active compounds in this series. Moreover, compound **16** has a higher score value and good inhibition as compared with its non-nitrated analog **15**. Therefore, the results suggest that the nitro group plays an important role in binding TcTS.

The binding mode B observed in this study differs from the DANA crystal structure (opposite binding conformation), and has not been previously described (Figure 5B). Binding mode B is associated with compounds **10**, **11**, **13**, **14**, **21** and **24** in their best-scored conformation; other compounds that exhibit binding mode B also have good scores, but not the best. Arg93 and Trp120 act as hydrogen donors to bind the carboxylate group at the position 1. These interactions can also be found involving a nitro group as a hydrogen acceptor. A variation of binding mode B was found where the amine group or derivatives at the position 4 form an interaction with Arg93 and Asp59. Figure 6C,D shows examples of compounds in binding mode B.

The third binding mode (C) did not show a specific binding conformation, and it was different from modes A and B. It is associated with compounds **20**, **22** and **23**; other compounds that exhibit binding mode C have good scores, but not the best. These compounds do not involve the characteristic interactions observed in the A and B modes or are binding on the exterior of the cavity (Figure 5C).

Table S1 (Supplementary Materials) shows the binding mode distribution for the best nine hits for each compound. It is noteworthy that some compounds exhibit two and even more binding modes with similar scores; therefore, the biological effect observed can be associated with several binding orientations.

## 3. Materials and Methods

### 3.1. Chemistry: General Procedure

Compounds **10** (4-aminobenzoic acid, A9878), **11** (4-hydrazinobenzoic acid, 246395), **13** (4-nitrobenzoic acid, 461091), **21** (4-amino 2-hydroxybenzoic acid, A79604), and **23** (2-hydroxybenzoic acid, 247588) were purchased from Sigma-Aldrich, Mexico city, Mexico, and used without further purification. In all compounds, synthesized melting points were determined on a Mel-Temp capillary apparatus (Electrothermal, Staffordshire, UK) and are uncorrected. Infrared spectra were recorded using a Bruker Alpha FT-IR spectrometer (AXS Inc., Madison, WI, USA). The ^1^H-NMR spectra were obtained in CDCl_3_ or DMSO-*d*_6_ with Me4Si as an internal standard on a Bruker Avance-300 Spectrometer operating at 400 MHz for ^1^H-NMR (AXS Inc., Madison, WI, USA). The purity and reactions were monitored by thin-layer chromatography (TLC) performed on silica gel plates prepared with silica gel 60 (PF-245 with gypsum, Merck, Tokyo, Japan), of the thickness of 0.25 nm. The developed chromatograms were visualized under ultraviolet light at 254–265 nm.

For *4-Azidobenzoic acid* (**12**): 4-aminobenzoic acid (**10**) (1.5 g, 10.9 mmol) was dissolved in HCl (15 mL) at 0 °C. The reaction mixture was stirred for 1 h, and 25 mL aqueous solution of NaNO_2_ (0.1 N) was added into the reaction mixture dropwise. The product was precipitated by adding the reaction mixture to the solution of CH_3_COONa (4.5 g, 61.3 mmol) and NaN_3_ (0.70 g, 10 mmol) in H_2_O (500 mL). The product was obtained by filtration and the residue was recrystallized in EtOH, yielding (**12**) (73%). IR (KBr): 2980 (CH); 2180 (N3); and 1680 and 1610 (C=O) cm^−1^. ^1^H-NMR (400 MHz, DMSO-*d*_6_) δ ppm: 7.1 (d, 2H, C_6_H_4_); 7.8 (d, 2H, C_6_H_4_); 12.1 (s, H, COOH). Calculated analysis for C_7_H_5_N_3_O_2_: C, 51.54; H, 3.09; N, 25.76. Found: C, 51.10; H, 2.75; N, 25.35.

*Ethyl 4-aminobenzoate* (**14**): 4-aminobenzoic acid (**10**) (3 g, 21.8 mmol) was dissolved in anhydrous ethanol (30 mL). Concentrated H_2_SO_4_ (1.0 mL) was added to the mixture and refluxed for 60 min. The reaction mixture was allowed to cool to room temperature, and the mixture was poured into 40 mL of ice water with continuous stirring. The mixture was neutralized by adding 15 mL of 10% Na_2_CO_3_. The white color precipitate was obtained, which was separated by vacuum filtration. The precipitates were washed with H_2_O, yielding (**14**) (90%). IR (KBr): 3410 and 3335 (NH_2_); 2984 (CH); 1678 and 1628 (C=O) cm^−1^. ^1^H-NMR (400 MHz, DMSO-*d*_6_) δ ppm: 1.3 (t, 3H, OCH_2_CH_3_); 4.1 (s, 2H); 4.3 (m, 2H, OCH_2_CH_3_); 7.1–7.2 (m, 2H, C_6_H_4_); 7.8–7.9 (m, 2H, C_6_H_4_). Calculated analysis for C_9_H_11_NO_2_: C, 65.44; H, 6.71; N, 8.48. Found: C, 65.30; H, 6.45; N, 8.15.

*Ethyl 4-acetamidobenzoate* (**15**): Ethyl 4-aminobenzoate (**14**) (1.5 g, 9 mmol) was added to a mixture (1:1) of acetic acid and acetic anhydride (20 mL), stirred, and the reaction mixture was refluxed for 15 min. After completion of the reaction, the mixture was poured into ice-cooled water and a solid residue was obtained after filtration. The crude was washed three times with 100 mL H_2_O to remove excess acid. The crude was recrystallized in EtOH, yielding (**15**) (86%). IR (KBr): 3332 (NH); 2984 (CH); 1680 and 1596 (C=O) cm^−1^. ^1^H-NMR (400 MHz, DMSO-*d*_6_) δ ppm: 1.29 (t, 3H, OCH_2_CH_3_); 2.1 (s, 3H, CH_3_); 4.29 (m, 2H, OCH_2_CH_3_); 7.70–7.72 (m, 2H, C_6_H_4_); 7.88–7.90 (m, 2H, C_6_H_4_); 10.27 (s, 1H, NH). Calculated analysis for C_11_H_13_NO_3_: C, 63.76; H, 6.32; N, 6.76. Found: C, 63.47; H, 6.05; N, 6.52.

For *4-Amino-3-nitrobenzoic acid* (**16**): 4-acetamido-3-nitrobenzoic acid (**17**) (2 g, 8 mmol) was taken in a reaction flask, H_2_SO_4_ (30 mL) was added dropwise with stirring for 15 min, and the mixture was heated for 15 min at 100 °C. After the completion of the reaction, the mixture was poured into ice-cooled water and a solid residue was obtained after filtration. The crude was washed three times with 100 mL H_2_O to remove excess acid. The crude was recrystallized in EtOH, yielding (**16**) (90%). IR (KBr): 3479 and 3362 (NH); 1622 (C=O); 772 (NO_2_) cm^−1^. ^1^H-NMR (400 MHz, DMSO-*d*_6_) δ ppm: 7.2 (s, 1H, C_6_H_3_NO_2_); 7.8 (s, 1H, C_6_H_3_NO_2_); 7.9 (s, 2H, NH_2_); 8.5 (s, 1H, C_6_H_3_NO_2_); 12.6 (s, 1H, COOH). Calculated analysis for C_7_H_6_N_2_O_4_: C, 46.16; H, 3.32; N, 15.38. Found: C, 45.89; H, 3.11; N, 15.08.

For *4-Acetamido-3-nitrobenzoic* acid (**17**): 4-acetamidobenzoic acid (**24**) (2.5 g, 14 mmol) was added slowly to a mixture (1:1) of HNO_3_ and conc. H_2_SO_4_ (40 mL) with stirring for 10 min at 0 °C. After, the mixture was stirred for 30 min at room temperature. The reaction mixture was poured into ice water and neutralized by adding 15 mL of 5% Na_2_CO_3_. The product was obtained by filtration and washed with an excess of H_2_O. The crude was purified by recrystallization with ethanol, yielding (**17**) (84%). IR (KBr): 3324 (NH); 2912 (CH); 1717 and 1672 (C=O); 773 (NO_2_) cm^−1^. ^1^H-NMR (400 MHz, DMSO-*d*_6_) δ ppm: 2.1 (s, 3H, CH_3_); 7.8 (s, 1H, C_6_H_3_NO_2_); 8.1 (s, 1H, C_6_H_3_NO_2_); 8.3 (s, 1H, C_6_H_3_NO_2_); 10.2 (s, 1H, NH); 12.6 (s, 1H, COOH). Calculated analysis for C_9_H_8_N_2_O_5_: C, 48.22; H, 3.60; N, 12.50. Found: C, 47.90; H, 3.30; N, 12.20.

*Ethyl 4-acetamido-3-nitrobenzoate* (**18**): Ethyl 4-acetamidobenzoate (**15**) (0.65 g, 3 mmol) and a mixture (1:1) of HNO_3_ and conc. H_2_SO_4_ (20 mL) were stirred and refluxed for 2 h. The reaction mixture was poured into ice water and neutralized by adding 15 mL of 5% Na_2_CO_3_. The product was obtained by filtration, washed with an excess of H_2_O, and the crude was purified by recrystallization with ethyl acetate, yielding (**18**) (75%). IR (KBr): 3357 (NH); 2991 (CH); 1712 and 1620 (C=O); 771 (NO_2_) cm^−1^. ^1^H-NMR (400 MHz, DMSO-*d*_6_) δ ppm: 1.33 (t, 3H, OCH_2_CH_3_); 2.12 (s, 3H, CH_3_); 4.31–4.37 (m. 2H, OCH_2_CH_3_); 7.85 (d, 1H, C_6_H_3_NO_2_); 8.20 (d, 1H, C_6_H_3_NO_2_); 8.36 (s, 1H, C_6_H_3_NO_2_); 10.56 (s, 1H, NH). Calculated analysis for C_11_H_12_N_2_O_5_: C, 52.38; H, 4.80; N, 11.11. Found: C, 52.10; H, 4.60; N, 10.80.

For *4-(4-Chlorobenzamido)-3-nitrobenzoic acid* (**19**): 4-amino-3-nitrobenzoic acid (**16**) (0.60 g, 3 mmol), 4-chlorobenzoyl chloride (0.84 g, 4 mmol), and Et_3_N (1.0 mL) were dissolved in dry CH_2_Cl_2_ (40 mL) and the reaction mixture was stirred for 48 h at room temperature. The mixture was filtered using a vacuum and the residue was washed thrice with 200 (mL) of H_2_O to remove the acid (HCl) produced during the reaction. The crude mixture was purified by column chromatography on silica gel in a CH_2_Cl_2_/EtOAc (3:1), yielding (**19**) (40%). IR (KBr): 3370 (NH); 2850 (CH); 1782 and 1683 (C=O) cm^−1^. ^1^H-NMR (400 MHz, DMSO-*d*_6_) δ ppm: 7.05 (d, 1H C_6_H_3_COOH); 7.5–7.6 (m, 2H, C_6_H_4_Cl); 7.83 (d, 1H, C_6_H_3_COOH); 7.87–7.89 (m, 2H, C_6_H_4_Cl); 7.9 (s, 1H, NH); 8.5 (s, 1H C_6_H_3_COOH); 12.8 (s, 1H, COOH). Calculated analysis for C_14_H_9_ClN_2_O_5_: C, 52.43; H, 2.83; N, 8.74. Found: C, 52.15; H, 2.56; N, 8.45.

For *4-(4-Methoxybenzamido)-3-nitrobenzoic acid* (**20**): 4-amino-3-nitrobenzoic acid (**16**) (0.60 g, 3 mmol), 4-methoxybenzoyl chloride (0.90 g, 4 mmol), and Et_3_N (1.0 mL) were dissolved in dry CH_2_Cl_2_ (40 mL) and the reaction mixture was stirred for 48 h at room temperature. The crude mixture was purified by column chromatography on silica gel in n-hexane/EtOAc (7:3), yielding (**20**) (24%). IR (KBr): 3338 (NH); 2965 (CH); 1786 and 1683 (C=O) cm^−1^. ^1^H-NMR (400 MHz, DMSO-*d*_6_) δ ppm: 3.89 (s, 3H, CH_3_); 7.13–7.16 (m, 3H: 2H, C_6_H_4_OCH_3_ and 1H, C_6_H_3_COOH); 7.98 (d, 1H, C_6_H_3_COOH); 8.07 (d, 2H, C_6_H_4_OCH_3_); 8.23 (s, 1H, NH); 8.67 (s, 1H, C_6_H_3_COOH); 12.8 (s, 1H, COOH). Calculated analysis for C_15_H_12_N_2_O_6_: C, 56.96; H, 3.82; N, 8.86. Found: C, 56.65; H, 3.45; N, 8.50.

For *4-Acetamido-2-hydroxybenzoic acid* (**22**): 4-amino 2-hydroxybenzoic acid (**21**) (1 g, 6.5 mmol) was added to a mixture (1:1) of acetic acid and acetic anhydride (20 mL), stirred, and the reaction mixture was refluxed for 15 min. After the completion of the reaction, the mixture was poured into ice-cooled water and a solid residue was obtained after filtration. The crude was washed three times with 100 mL H_2_O to remove excess acid. The crude was recrystallized in EtOH, yielding (**22**) (85%). IR (KBr): 3326 (NH); 2912 (CH); 1720 and 1680 (C=O) cm^−1^. ^1^H-NMR (400 MHz, DMSO-*d*_6_) δ ppm: 2.1 (s, 3H, CH_3_); 7.6 (s, 1H, C_6_H_3_OH); 7.8 (s, C_6_H_3_OH); 8.1 (s, C_6_H_3_OH); 10.21 (s, 1H, NH); 12.6 (s, 1H, COOH). Calculated analysis for C_9_H_9_NO_4_: C, 55.39; H, 4.65; N, 7.18. Found: C, 54.95; H, 4.10; N, 6.85.

For *4-Acetamidobenzoic acid* (**24**): 4-aminobenzoic acid (**10**) (4 g, 29 mmol) was added to a mixture (1:1) of acetic acid and acetic anhydride (20 mL), stirred, and the reaction mixture was refluxed for 15 min. After the completion of the reaction, the mixture was poured into ice-cooled water and a solid residue was obtained after filtration. The crude was washed three times with 100 mL H_2_O to remove excess acid. The crude was recrystallized in MeOH, yielding (**24**) (90%). IR (KBr): 3340 (NH); 2539 (CH); 1700 and 1607 (C=O), cm^−1^. ^1^H-NMR (400 MHz, DMSO-*d*_6_) δ ppm: 2.08 (s, 3H, CH_3_); 7.68 (d, 2H, C_6_H_4_); 7.87 (d, 2H, C_6_H_4_); 10.24 (s, 1H, NH); 12.68 (s, 1H, COOH). Calculated analysis for C_9_H_9_NO_3_: C, 60.33; H, 5.06; N, 7.82. Found: C, 59.80; H, 4.90; N, 7.52.

### 3.2. Biological Assays

#### 3.2.1. Trypanocidal Activity

In vitro studies were carried out using two strains of trypomastigotes of *Trypanosoma cruzi*: NINOA and INC-5. CD-1 Mice (18–20 g) were inoculated intraperitoneally with 1 × 10^6^/mL of blood trypomastigotes (0.2 mL). Blood was obtained by cardiac puncture of mice infected with trypomastigotes at the peak of parasitemia, using heparin as an anticoagulant. Blood was treated with isotonic saline (NaCl 0.85%) to adjust to a concentration of approximately 2 × 10^6^ trypomastigotes/mL and 195 µL of blood and 5 µL of treatment was placed in 96-well plates. Treatments consisted of a negative control, containing dimethyl sulfoxide (DMSO 2.5%) and compounds derived from benzoic acid, and nifurtimox and benznidazole dissolved in DMSO at the following concentrations: 200 µg/mL, 100 µg/mL, 50 µg/mL, 25 µg/mL, and 12.5 µg/mL, to get the lysis concentration of 50% of the population (LC_50_). LC_50_ values were determined using a Probit statistical analysis of the dose-response, and the results are expressed as the mean ± standard deviation. Finally, crystal violet (1 µg/mL) for the lysis of the trypomastigotes and as witness wells containing 200 µL of blood without receiving any treatment were used. Each concentration was tested in triplicate.

Once the compounds derived from benzoic acids after being homogenized with the blood were added, the plates were incubated at 4 °C for 24 h. After the incubation, the plates were kept at room temperature for 30 min and then an aliquot of 5 mL of each well was taken, which was placed between a slide and a coverslip, and viable trypomastigotes were counted using the method of Brener [51] supplemented with Pizzi. The results were later converted to micromolar units.

#### 3.2.2. Enzymatic Inhibition Assays

Inhibition was assessed using the continuous fluorimetric assay described by Douglas and co-workers [52]. The assay was performed in triplicate (and on three different days) in 96-well plates containing phosphate buffer solution at pH 7.4 (25 µL), a recombinant enzyme solution (25 µL), and an inhibitor solution (25 µL of a 4.0 mM solution). This mixture was incubated for 10 min at 26 °C followed by the addition of MuNANA (Km = 0.68 mM; 25 µL of a 0.4 mM solution giving an assay concentration of 0.1 mM). The final concentration of the tested compounds was 1.0 mM, and the positive control was pyridoxal phosphate. The fluorescence of the released product (Mu) was measured after 10 min, with excitation and emission wavelengths of 360 and 460 nm, respectively, and the data were analyzed with GraphPad Prism software version 4.0 (San Diego, CA, USA). Inhibition percentages were calculated by the equation: % I = 100 × [1 − (*V_i_*/*V*_0_)], where *V_i_* is the velocity in the presence of the inhibitor, and *V*_0_ is the velocity in the absence of the inhibitor.

#### 3.2.3. Molecular Docking

Ligand preparation: Compounds **10**–**24** were built in Maestro 9.1 and their geometry was optimized using the universal force field (UFF) [53,54]. Then, the ligands were exported to AutoDock Tools 1.5.6 in .pdb format to generate .pdbqt files [50,55,56].

Protein preparation: The TcTS were obtained from the Protein Data Bank with the PDB accession number 1MS8 [53]. The structure was prepared using Maestro 9.1 [56], first, the chain A was selected and the ligands, solvent, and other molecules were removed. Missing side chains were added and alternative side chains were defined using Maestro. Then, the .pdb structure was submitted to minimization using the YASARA web server [57]. The optimized structure was exported to AutoDock Tools 1.5.6 in .pdb format to generate a .pdbqt file.

Docking studies: AutoDock Vina 5.6 was used to predict the mode of interaction for each ligand within the active site of TcTS [58]. A grid box of x, y, and z dimensions was set to 60, 60, 60 angstroms centered to the DANA binding site in the original crystal structure. Each compound was set to run 100 dockings, and the best nine conformations were retrieved for the analysis. The results were analyzed employing PyMOL v0.99 and UCSF Chimera 1.11rc [59].

## 4. Conclusions

In this work, the benzoic acid derivatives **14**, **18** and **19** clearly showed more potent trypanocidal activity than the commercially available drugs beznidazole and nifurtimox towards the NINOA and INC-5 strains of *T. cruzi*. It is noteworthy that compound **18** showed nanomolar trypanocidal activity against the NINOA strain (20 nM), whereas compounds **11** and **17** displayed similar TcTS inhibition to pyridoxal and compound **16** showed the best inhibitory activity.

TcTS inhibition assays provided evidence that the *p*/*m*-nitrobenzoic acid cores (**13**, **16**–**18**) and the *p*-hydrazine benzoic acid (**2**) are relevant for TcTS inhibition, whilst trypanocidal assays against the NINOA and INC-5 strains showed higher anti-parasite activity for the *p*-aminobenzoate derivative compounds (**14**–**15**) and the *p*-amino-*o*-hydroxylbenzoic acid compound **21** via alternative mechanisms. The ethyl benzoate compounds **14**, **15**, and **18** displayed higher trypanocidal activities than their precursors (**10**, **24**, and **17**, respectively) but a reduced inhibition of TcTS.

The respective docked structures of the compounds showed three different binding patterns according to DANA crystal structure in the active site cavity. Model A is similar to DANA interaction in the cavity, model B represents the opposite binding conformation, and model C is interactions outside the cavity or that do not involve the characteristic interactions observed in the A and B modes.

The benzoic acid derivatives (**10**–**24**) evaluated in the present work for the treatment of Chagas disease by the inhibition of TcTS or alternative biological mechanisms (trypanocidal activity) reinforce the development of more effective candidates of this disease. Therefore, we suggest ethyl 4-acetamido-3-nitrobenzoate **18** as a prototype for the development of more effective TcTS inhibitors against Chagas disease, which shows a moderate inhibition (47%), a binding model similar to DANA (pattern A), and significant trypanocidal activity (LC_50_ values of 0.02 and 0.22 µM against the NINOA and INC-5 strains, respectively).

## Figures and Tables

**Table 1:**
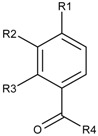
Trypanocidal activity of benzoic acid derivatives on the NINOA and INC-5 strains.

Code	R_1_	R_2_	R_3_	R_4_	NINOA LC_50_ (µM)	INC-5 LC_50_ (µM)
**10**	NH_2_	H	H	OH	0.52 ± 0.19	1.24 ± 1.0
**11**	NHNH_2_	H	H	OH	0.66 ± 0.39	0.58 ± 0.4
**12**	N_3_	H	H	OH	0.60 ± 0.46	0.47 ± 0.35
**13**	NO_2_	H	H	OH	0.47 ± 0.16	0.46 ± 0.38
**14**	NH_2_	H	H	OCH_2_CH_3_	0.10 ± 0.041	0.10 ± 0.047
**15**	NHCOCH_3_	H	H	OCH_2_CH_3_	0.34 ± 0.18	0.21 ± 0.1
**16**	NH_2_	NO_2_	H	OH	1.37 ± 0.56	0.63 ± 0.3
**17**	NHCOCH_3_	NO_2_	H	OH	1.10 ± 0.58	0.21 ± 0.1
**18**	NHCOCH_3_	NO_2_	H	OCH_2_CH_3_	0.02 ± 0.012	0.22 ± 0.09
**19**	NHCOC_6_H_4_-*p*-Cl	NO_2_	H	OH	0.14 ± 0.08	0.0008 ± 0.0001
**20**	NHCOC_6_H_4_-OCH_3_	NO_2_	H	OH	0.61 ± 0.3	0.43 ± 0.28
**21**	NH_2_	H	OH	OH	0.27 ± 0.10	0.26 ± 0.09
**22**	NHCOCH_3_	H	OH	OH	1.28 ± 026	1.28 ± 0.31
**23**	H	H	OH	OH	0.576 ± 0.32	0.721 ± 0.42
**24**	NHCOCH_3_	H	H	OH	1.39 ± 0.75	0.878 ± 0.55
**Nfx**					0.213 ± 0.08	0.68 ± 0.17
**Bzn**					0.292 ± 0.12	0.62 ± 0.28

LC_50_: lysis concentration of 50% of the population.

**Table 2:**
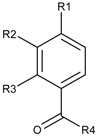
TcTS inhibition values of benzoic acid derivatives.

Code	R1	R2	R3	R4	% Inhib. at 1 mM
**10**	NH_2_	H	H	OH	30
**11**	NHNH_2_	H	H	OH	61
**12**	N_3_	NO_2_	H	OH	40
**13**	NO_2_	H	H	OH	43
**14**	NH_2_	H	H	OCH_2_CH_3_	1
**15**	NHCOCH_3_	H	H	OCH_2_CH_3_	7
**16**	NH_2_	NO_2_	H	OH	77
**17**	NHCOCH_3_	NO_2_	H	OH	66
**18**	NHCOCH_3_	NO_2_	H	OCH_2_CH_3_	47
**19**	NHCOC_6_H_4_-*p*-Cl	NO_2_	H	OH	Not tested *
**20**	NHCOC_6_H_4_-*p*-OCH_3_	NO_2_	H	OH	Not tested *
**21**	NH_2_	H	OH	OH	32
**22**	NHCOCH_3_	H	OH	OH	34
**23**	H	H	OH	OH	17
**24**	NHCOCH_3_	H	H	OH	30
**Pyr**					64

* Not tested due to low solubility. The standard deviation for each experiment was <5%.

## Supplementary Materials

The following are available online. Table S1: Binding modes and docking scores for compounds **10**–**24**.
