# Supplementary material for: Benzoic Acid Derivatives with Trypanocidal Activity: Enzymatic Analysis and Molecular Docking Studies toward Trans-Sialidase
Source: Molecules. 2017 Oct 30;22(11):1863. doi: 10.3390/molecules22111863 (PMC6150317; doi:10.3390/molecules22111863)
Supplement: Supplementary file 1 [file molecules-22-01863-s001.pdf]

Supplementary material

Table S1. Binding modes and docking scores for compounds **10**–**24**

Table S1. Binding modes and docking scores for compounds **10**–**24**

	**Lowest energy conformation**		**Binding mode for the top nine low energy conformations**					
**Code**	**Bind**	**Score**	**Bind A (A2)[1]**	**Bind B**	**Bind C**	**Max. Score[2]**	**Min. Score[3]**	**Mean Score[4]**
**10**	B	-5.8	1	6	2	-5.8	-4.9	-5.3
**11**	B	-6.3	1	6	2	-6.3	-5.6	-5.9
**12**	A2	-6.9	4 (3)	0	5	-6.9	-6.2	-6.5
**13**	B	-5.9	2 (2)	2	5	-5.9	-5.7	-5.8
**14**	B	-6.0	1	4	4	-6.0	-5.1	-5.6
**15**	A	-6.8	3	0	6	-6.8	-5.7	-6.1
**16**	A2	-6.3	4 (2)	0	5	-6.3	-5.9	-6.1
**17**	A2	-7.4	5 (3)	0	4	-7.4	-6.5	-6.8
**18**	A2	-7.4	5 (3)	0	4	-7.4	-6.5	-7
**19**	A2	-8.0	1 (1)	0	8	-8.0	-6.9	-7.4
**20**	C	-7.8	0	1	8	-7.8	-6.7	-7.1
**21**	B	-5.9	1	3	5	-6.9	-6.4	-5.5
**22**	C	-6.9	2	2	5	-6.9	-6.4	-6.6
**23**	C	-5.8	0	2	7	-5.8	-5.1	-5.4
**24**	A, B	-6.3	2	1	6	-6.3	-5.8	-6.0

[1] Represents the population from the top nine conformations with binding mode A.
[2] Maximum score found in the top nine conformations.
[3] Minimum score found in the top nine conformations.
[4] Mean score found in the top nine conformations.
